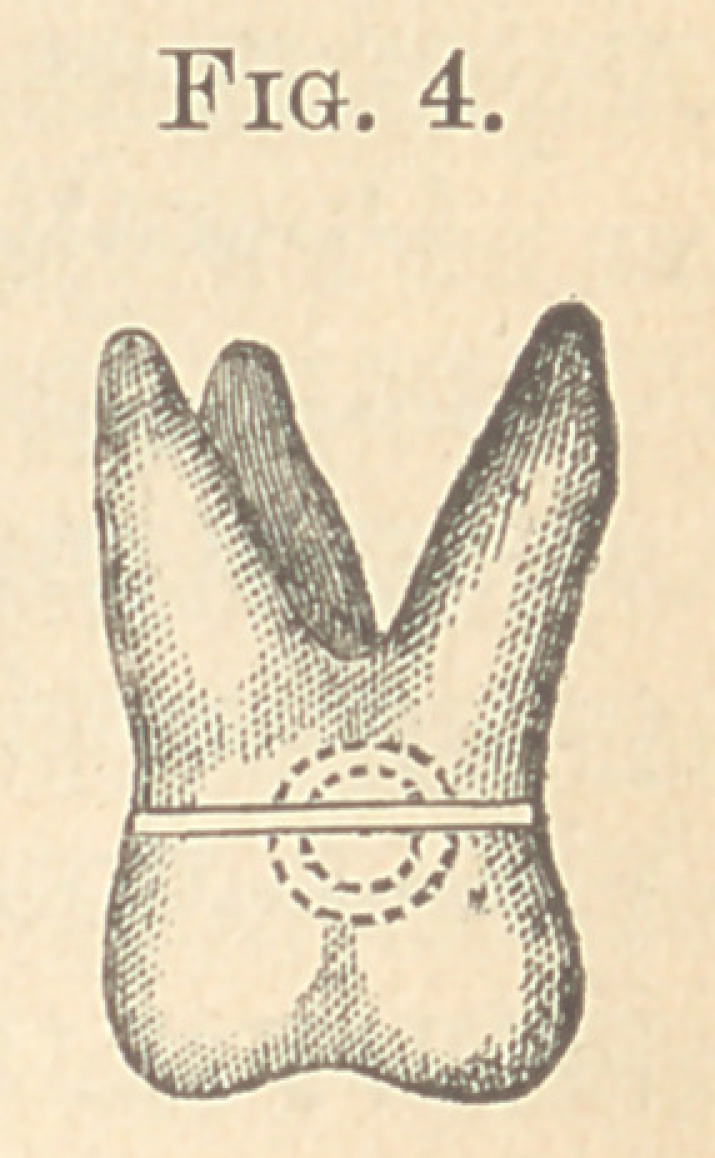# A Suggestion

**Published:** 1889-10

**Authors:** Theodore F. Chupein

**Affiliations:** Philadelphia


### A SUGGESTION.

BY THEODORE F. CHUPEIN, D.D.S., PHILADELPHIA.

THE following suggestion regarding natural tooth crown substitutes may meet an emergency in some cases. The idea occurred to me, but I have not had a case thus far to test its feasibility. I term it the "natural crown," as it is made from the crown of a natural human tooth. It sometimes happens that good teeth are extracted, which are free from any trace of decay, to relieve a crowded condition of the dental arch; but even should the crowns be decayed, either slightly or extensively, this will be no impediment to the operation, as the decayed places may be filled or contoured either with gold or amalgam, and serve as substitutes for lost tooth crowns.

For facility of description we will take a typical case,—an upper molar. The remains of the crown are filed or ground down flat or level, a little above the margin of the gum. The roots are seamed out, treated and filled, and the pulp-chamber prepared as shown at Fig. 1. The crown of an upper molar, of the proper size and side, is selected, filled, and contoured in its decayed places, if the selected tooth has decayed places, and sawed off at its neck. The pulp-chamber of the crown is likewise prepared by properly seaming and undercutting with suitable engine-burrs.

FIG. 1.

Such of the natural teeth as come into our hands by extractions should be kept submerged in *strong brine*, or perhaps preferably in a jar of alcohol. If permitted to become dry after extraction, they get so brittle that little can be done with them in cutting, scraping, or seaming them for filling.

When the root and crown are prepared, as described, the pulp-chamber in the crown is filled with wax, so that a little of the wax protrudes from it, sufficient to fill the pulp-chamber in the root. With the help of the wax to hold the crown in position the crown may be properly articulated by grinding away with corundum wheels in the engine, at such points as are indicated by the occlusion. This being accomplished, the crown is filed down at its neck, about the thickness of cardboard, or a trifle more, for reasons that will become apparent. A piece of eighteen carat gold plate, about twenty-eight or thirty gauge thickness, and of a size that will amply cover the neck of the crown is punched or drilled at points about one-thirty-second of an inch apart, as shown at Fig. 2. These holes are then countersunk. A piece of wire, bent like a staple, is passed through these holes and soldered to the plate, with two minute pieces of solder just sufficient to fill the countersunk holes, the protruding ends of the wire are bent so as to form another staple on the other side of the plate, and soldered also, as shown at Fig. 3. The plate, as thus constructed, is filed to the proper size, as indicated by the size of the neck of the crown or root. The root is now well dried, and the proper provisions made for the exclusion of moisture. The pulp-chamber of the root is filled with zinc, phosphate, cement, and a little of the same placed within the loop on the plate. The plate is then placed on the root and kept in close contact and proper position, until the cement hardens. The pulp-chamber, in the crown, is then filled with cement, and a little placed within the loop on the plate, when it is carried to its position. After the cement has set, the operation is completed by scraping off such small portions of the cement as may have oozed through between the root and plate and between the crown and plate. There is nothing to show that this substitute differs from a natural tooth-crown except the line of the gold showing the plate which binds the two parts together, as at Fig. 4. The dotted lines indicate the staples on the plate within the pulp-chamber.

## Figures and Tables

**Fig. 1. f1:**
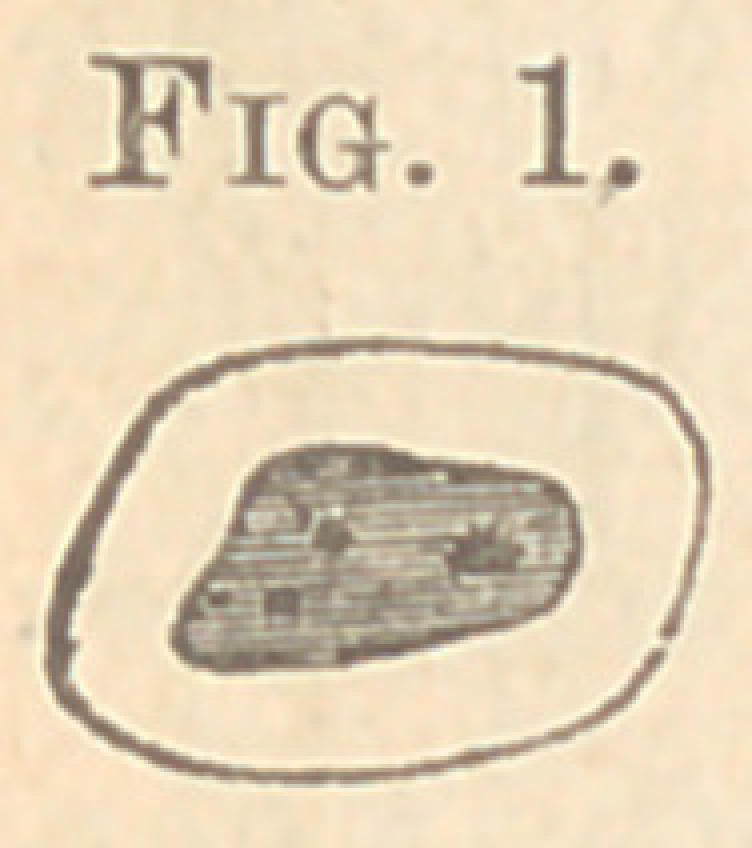


**Fig. 2. f2:**
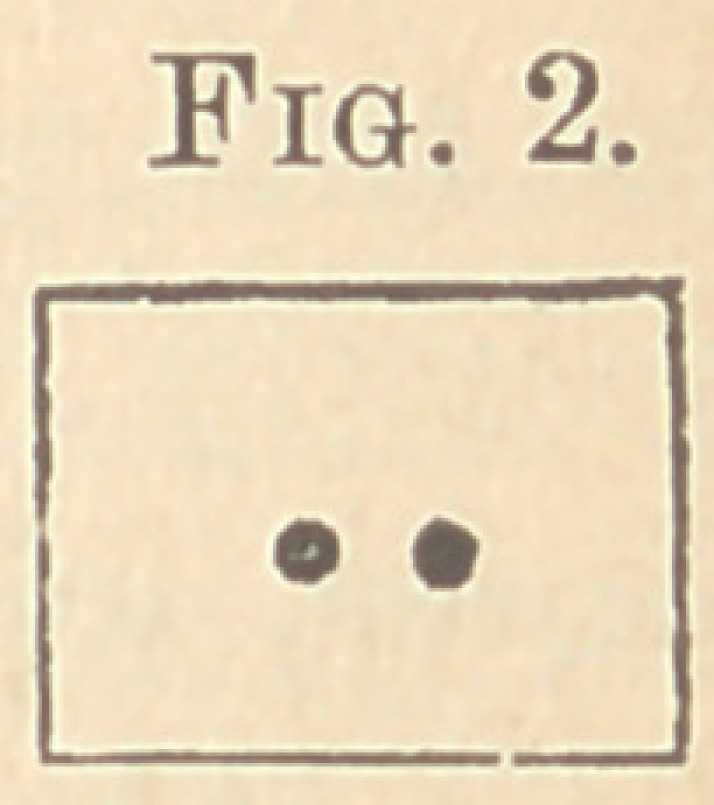


**Fig. 3. f3:**
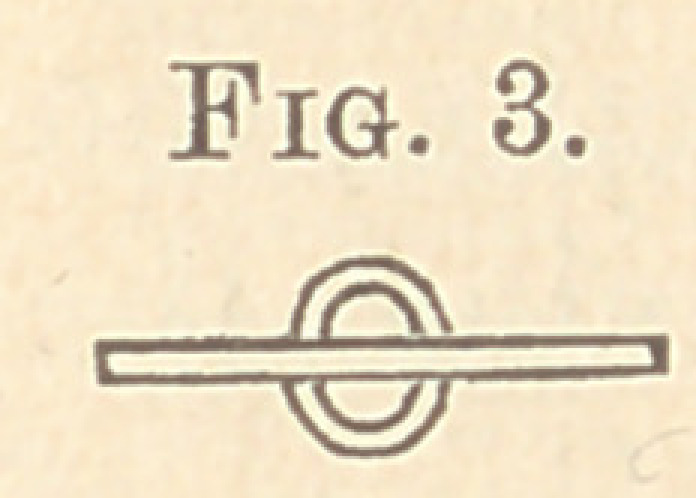


**Fig. 4. f4:**